# Access to and Use of Health Services by Older Men and Women Experiencing Frailty and Ageing in Place Alone in Italy

**DOI:** 10.3390/healthcare13212684

**Published:** 2025-10-23

**Authors:** Maria Gabriella Melchiorre, Marco Socci, Giovanni Lamura, Sabrina Quattrini

**Affiliations:** Centre for Socio-Economic Research on Ageing, IRCCS INRCA—National Institute of Health and Science on Ageing, Via Santa Margherita 5, 60124 Ancona, Italy; g.melchiorre@inrca.it (M.G.M.); g.lamura@inrca.it (G.L.); s.quattrini@inrca.it (S.Q.)

**Keywords:** ageing in place, older people experiencing frailty, functional limitations, chronic diseases, drugs, health services, general practitioners, barriers to use services, gender, Italy

## Abstract

**Background**: Access to and use of health services represent crucial issues/challenges for older people experiencing frailty with functional limitations and chronic diseases, especially when they age in place alone. Both access to and use of health services are also characterised by gender differences. The present study analysed these factors in three Italian regions (Lombardy, North; Marche, Centre; and Calabria, South), where in 2019, the “Inclusive Ageing in Place” (IN-AGE) project was carried out, involving 120 senior people aged 65 years and of both genders. **Methods**: In this mixed-methods study, both qualitative (predominant section) and some quantitative data (e.g., socio-demographic aspects and functional limitations) were collected through semi-structured interviews. In addition to basic quantitative analyses, content analysis and the quantification of statements were performed to process the qualitative data. The results for both men and women are presented. Possible barriers to accessing health services were also considered. **Results:** Women reported more cases of chronic diseases than men, especially arthritis/osteoporosis, and a greater use of drugs than men. Both genders used services provided by the general practitioner (GP) and medical specialist (MS), the latter being mostly private. More women than men used rehabilitation, especially in the private sector, and reported the issue of cost for private healthcare and the travel distance to reach medical units as barriers to access. The long waiting lists/times were complained about by both males and females. **Conclusions**: This study, despite its simple/descriptive qualitative approach with a limited sample, could provide, however, some insights for policymakers and healthcare professionals to plan prevention policies and deliver appropriate and timely health services to older people experiencing frailty and ageing in place alone, devoting attention to gender-related issues in the design and provision of such services.

## 1. Introduction

The possibility of ageing in place, i.e., remaining in one’s own home and being integrated into local communities for as long as possible, thus maintaining one’s own memories [1,2,3], as opposed to moving to a nursing home, is reported as the preferred housing solution by the majority of older people [4,5,6]. In Europe, 50% of women and 25% of men aged 75 and over live alone [7], and these numbers are 50% and 22%, respectively, in Italy [8]. The large proportion of senior people living alone is characterised by some changes having occurred in their family structure, i.e., a lower number of children and decreased availability of caring relatives, with increased pressure on long-term care (LTC) [9].

Ageing alone at home, without cohabiting relatives, is particularly challenging for senior people experiencing frailty with functional limitations, since these could limit/hamper daily activities [3]. Indeed, frailty is a multidomain concept, involving demographic aspects, lifestyle, multimorbidity, neighbourhood relations, social participation, economic status, and available social support [10,11,12]. Interestingly, Fried et al. [13] developed and operationalised a standardised phenotype of frailty in community-dwelling older people, defined as a clinical/physiologic syndrome, that includes, among other factors, unintentional weight loss, exhaustion, and weakness. In particular, within a physiologic cycle of frailty, they identified an intermediate stage (with one or two frailty characteristics), regarding senior people at high risk of frailty, between those experiencing this condition and those who were not, with comorbidities representing an etiologic risk and disability being an outcome. With regard to gender, older women in European countries exhibit greater functional limitations than men [14] in both basic (e.g., personal care) and instrumental (e.g., housework) activities of daily living (ADLs and IADLs) [15]. In Italy, in 2019, these values were 13% vs. 7% and 35% vs. 18%, respectively [16]. More recent data regarding older people aged 65 years and over in 2023 [17] revealed that 49% of men and 54% of women in the European Union (EU-27) and 43% and 52% in Italy reported long-standing limitations due to health problems.

Health thus represents a crucial issue in later life, with people living longer but in worse conditions and requiring increasing care and solutions [9]. Specifically, multimorbidity, i.e., the co-occurrence of multiple long-term health conditions/chronic diseases (two and more) in an individual [18], is strictly associated with the ageing process, and its prevalence in the EU-27 ranges from 30% among those aged 45–64 years to 82% among people aged 85 years and over [9]. In particular, 44% of senior people aged 65 years and over reported two chronic diseases (on average in the EU-27 in 2021–2022), with a greater prevalence for women than for men (46% vs. 40%) [19]. Chowdhury et al. [20], in their worldwide systematic review and meta-analysis, reported that 51% of adults aged over 60 years suffer from multimorbidity, with a prevalence that differs by age and gender and with higher values observed in senior people and females. Multimorbidity includes heart/cardiovascular disease, hypertension, arthritis, chronic respiratory disease/asthma, diabetes, stroke, cancer, Alzheimer’s disease, and depression [21]. In particular, diabetes, hypertension, and heart/respiratory failure affect 80% of senior people worldwide [22]. Osteoarthritis represents a crucial cause of disability in Europe, also following the rapid ageing of the population. Heart/circulatory diseases and cancer are the main causes of mortality, accounting for 32% and 22%, respectively, of all deaths in 2021 [19]. A more recent source, reporting the main causes of death by gender in 53 Member States of the WHO European Region in 2024 [23], indicates a greater prevalence of ischemic heart disease and stroke in females and of lung cancer and chronic obstructive pulmonary disease (COPD) in males. However, for older people aged 70 years and over of both genders, ischemic heart disease represents the most important cause of death. A previous wide systematic analysis of the Global Burden of Disease Study carried out in 2021 across 204 countries in seven world regions [24] found health differences between women and men in the period 1990–2021, which are constant throughout the course of life and increase with ageing, with higher morbidity among females, especially with regard to musculoskeletal and depressive disorders.

In Italy, in 2019, 43% of senior people aged 65 and over reported at least one chronic disease, and 17% reported at least two. Also, the most common diseases were osteoarthritis and hypertension (approximately 50% each) [16]. According to the country health profile for Italy in 2023, as reported by the OECD [25], the proportion of people aged 65 and over with multiple chronic conditions was 20% for men and 35% for women. Thus, women have higher levels of multimorbidity and worse self-rated health (SRH) than men, even though the former have a longer life expectancy at 65 years (EU-27: 21 vs. 17 years; Italy: 22 vs. 19 years) [9].

Multimorbidity and frailty also imply the increased use of health services [26] and related high healthcare expenditures, with costs rising from 54% to 101% if, respectively, three or four/five frailty symptoms (i.e., weakness, slowness, exhaustion, unintentional weight loss, and physical inactivity) are reported, as emerged in a study carried out in Germany [27]. Additionally, about 80% of global/world health resources are estimated to be necessary to address chronic care [22], and polypharmacy, i.e., multiple medications, is linked to older people with multimorbidity, who also consume five or more drugs daily [28]. In Italy, 46% of hospital admissions and 60% of pharmaceutical expenditure involve older people aged 65 years [29]. Also, according to ISTAT [16], in the four weeks preceding the interview for a survey on the health conditions of senior people in Italy, 90% and 66% of them, respectively, had a visit from a general practitioner (GP) and from a medical specialist (MS), and 14% used professional rehabilitation. Notably, in 2021, Italy’s health expenditures accounted for 9.4% of its gross domestic product (GDP), which is lower than the EU-27 average (11%) [25]. This context is crucial for older women, whose health conditions are worse than those of older men, both in Europe and Italy. This leads the former to need and use more health services (about two to five percentage points more than men), especially rehabilitation, diagnostic tests, GPs, and MSs. Also, the use of drugs is reported to be greater for older Italian women than for men (84% vs. 75%) [16,30].

It is worth specifying that in Italy, public primary care is provided by GPs, who act as gatekeepers for accessing higher/specialised levels of care, and formally provide patients with the necessary documentation for using public health services. Italy has a National Health Service (NHS), which works on a regional basis and provides universal health coverage to all citizens and registered foreign residents (those not registered can access urgent/essential health services). The central government provides regions with public funds and defines essential levels of care, whereas regions deliver services (primary, specialist, and hospital care) [25,31]. Health services are largely free of charge, even though a co-payment is requested in some cases. However, the cost of public health services is free (without the payment of a ticket) for children under 6 and senior people over 65 years of age living in households with incomes lower than 36,000 EUR. Further exemptions (for some or all services) are assured, e.g., to individuals affected by some chronic/rare diseases, those with a validated disability status, and pregnant women [31]. Citizens may also receive healthcare (e.g., hospitals, MSs, specialist ambulatory services) from privately accredited providers funded by public resources, from private insurance companies, or they can purchase private health services directly from for-profit organisations, using their own financial resources [31,32].

For older people experiencing frailty who are ageing in place alone, with multimorbidity and functional limitations, possible barriers preventing/making the access to and use of healthcare services difficult, both in the public and private sectors, become public health issues. Too many obstacles can even lead to giving up treatments. According to some authors [31], in 2019, about 2% of Italian residents (not only senior people) reported unmet health needs, due to long waiting times/lists and unaffordable requests for co-payment for public services. Other authors reported long waiting lists in the public sector and high costs in the private sector as the main barriers for senior people in accessing these services [33]. ISTAT data for Italy [16] indicated that, in 2019, the main barriers to using health services were long waiting lists (20%); however, economic reasons (10%) were also important factors. This applied more to older women than older men (19% vs. 14%). Further ISTAT data [30] similarly reported the same obstacles to medical care faced by older Italian people, as well as transportation problems, for women especially (8%), and difficulty accessing buildings due to architectural barriers (about 4%). This picture also applies to European countries more generally. According to EUROSTAT [34], in 2018, in the EU-27, the three main reasons leading to unmet health needs and related medical examinations were the high cost of medical services, long waiting lists, and medical units that are “too far to travel”.

The context described above highlights crucial consequences for health and daily living, especially for older women ageing in place alone. In Italy, we explored these factors by means of interviews with the aim of capturing the concrete experiences of senior people, from the exigent perspective of needing to understand such issues in more depth and to integrate available statistics with narratives, providing information on different personal attitudes and expressions, thus obtaining added knowledge on the topic. In this respect, this study used findings from the “Inclusive Ageing in Place” (IN-AGE) research project [35] to answer the following research questions regarding older people aged 65 years and over living alone in Italy: (1) What are the main diseases suffered by senior people experiencing frailty, and what drugs do they use? (2) What are the main public and private health services used? (3) What are the main barriers to accessing and using health services? (4) Are there gender differences with regard to diseases, the use of drugs, and the use of health services? According to the available literature and data reported above, it can be seen that older women suffer from more diseases and use more drugs. Also, they use a variety of different health services (mainly in the private or public sector) and face greater barriers (e.g., cost of private services, combined with the female economic disadvantage).

The findings from this study could provide useful insights for policymakers and healthcare professionals for developing prevention policies, in addition to appropriate cure–care strategies, and allow the implementation of adequate and timely health services for older people experiencing frailty, taking into account gender-related issues.

## 2. Materials and Methods

### 2.1. Setting and Sampling

**Figure 1:**
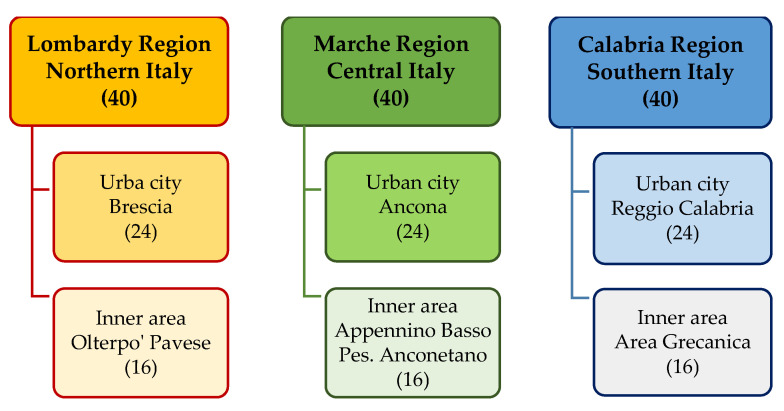
Regions and urban–rural sites. The number of interviews carried out at each territorial level (region, urban city, inner area) are reported in brackets.

The IN-AGE survey was carried out from May to December 2019 in Lombardy, Marche, and Calabria. These are three Italian regions which are considered representative of diverse levels of socio-economic development and service provision, which are, respectively, high in the north, middling in the centre, and low in the south of the country [36] (Figure 1).

In each region, 40 older people were recruited (for a total of 120 individuals—90 women and 30 men; the recruitment process is better described in Section 2.2 below) from one medium-sized urban city with about 100,000–200,000 residents (respectively, Brescia, Ancona, and Reggio Calabria) and one rural/inner area (respectively, Oltrepò Pavese, Appennino Basso Pesarese e Anconetano, and Area Grecanica) (Figure 1).

In particular, rural/inner areas are those defined by the National Strategy for Inner Areas [37] as zones hard to access/reach, with a notable ageing process and socio-economic depression, in addition to a poor provision of healthcare services. The respective rural municipalities of each inner area are as follows: Menconico, S. Margherita di Staffora, and Varzi from Oltrepò Pavese; Apecchio, Cagli, and Piobbico from Appennino Basso Pesarese Anconetano; and Roccaforte del Greco and San Lorenzo from Area Grecanica.

A purposive (non-probability) sample was created, and participants were included for their characteristics, allowing the adequate exploration of the study topics, rather than for their statistical representativeness [38]. The criteria for including old individuals experiencing frailty were as follows: men and women aged 65 years and over; living alone at home, or at least with a personal/private care assistant (PCA); mobility ranging from being limited within the home to going outside the home with help; no cognitive impairment to be able to answer questions independently; and the absence of support from family members living in the same urban block/rural building [35]. In our study, a simplified definition of frailty was thus adopted, which implies only older persons living alone, with physical decline, functional limitations/reduced independence, and consequent need for support to perform both ADLs and IADLs [39].

### 2.2. Ethical Considerations, Recruitment Process of Participants, and Data Collection

Before the data collection began, this study was approved by the Ethics Committee of the Polytechnic of Milan (POLIMI, Research Service, Educational Innovation Support Services Area), authorisation n. 5/2019, on 14 March 2019. This study was also conducted in accordance with the ethical guidelines outlined by the European General Data Protection Regulation (GDPR) n. 679 of 27 April 2016 [40].

Participants were recruited with the support of the local sections of voluntary associations (e.g., Auser, Anteas, Caritas) and operators of public home care/social services. These first checked the eligibility of senior people, especially with regard to their cognitive status and physical autonomy, on the basis of regular assessments carried out by these associations/service providers. These aspects were, however, further confirmed/validated by the interviewers (e.g., psychologists) when speaking with the families/caregivers of older respondents to schedule the appointments for the interviews. The contact details of the senior people who met the inclusion criteria and expressed general/preliminary verbal consent to participate were passed to the research teams. Those who agreed to be interviewed, after receiving exhaustive information on the aim and procedure of the survey, signed a written informed consent form containing detailed information on the study protocol, including the confidentiality and anonymity of their personal information.

With regard to the participation rate, 58% of the available senior people were included in this study (120 out of 208), whereas 42% declined due to illness/hospitalisation or reconsideration during the survey. With respect to data saturation, recruitment was stopped when, by reading the 120 transcripts step-by-step, additional categories/codes generated from the interviews did not add relevant information beyond what was already gathered. Saturation was thus set at the stage of data collection “as a matter of identifying redundancy in the data” [41] (p. 1896) and at a “degree to which new data repeat what was expressed in previous data” [41] (p. 1897). In particular, we adopted the “code meaning” approach, by assessing both code saturation, i.e., the sufficiency of newly identified issues since “we heard it all” [42] (p. 591), and meaning saturation, i.e., the sufficiency of nuances/insights already gathered to “understand it all” [42] (p. 591). Thus, the meaning of codes was further captured in addition to counting the prevalent codes [42]. The data collected with 120 interviews indicated that the information gathered was congruous with the topic of this study and the research questions, and how various typologies of frail senior people which emerged were adequately illustrative/characteristic of the target population. For instance, the greater proportion of older women than men with functional limitations and widows, as confirmed in previous studies [16], was respected, in addition to a low proportion of senior people with a PCA, who usually assist older people in worse physical/cognitive condition. In our sample, we included an intermediate level of mobility (from mainly remaining in the home to being able to go outside with help at least two times a week), but excluded senior people who were confined to beds and presented cognitive impairment [35].

Data collection was provided by three psychologists and three sociologists (five females and one male, two interviewers in each region) with in-depth expertise in research involving older people. Face-to-face interviews were conducted at the homes of senior people and lasted approximately 60–90 min. In 32 cases, senior people requested the presence of a son or a daughter during the interview for greater peace of mind (as reported by the interviewers). Also, when a senior person was supported by a PCA (27 cases), the latter was present at certain points. The transcripts were not returned to senior participants for comment and/or validation of insights from the narratives in order to restrict possible concerns/hardships linked to their involvement in the research. A brief pilot test was carried out in each region (one test per region), to refine the preliminary protocol; this was the first version of this document and comprised study design, methodology, recruitment, and topic guide. This was also proposed as general information for the potential participants. Following this pilot, and according to the critical recruitment situations preliminarily emerging in some areas, it was deemed appropriate to slightly broaden the selection criteria, which was implemented in the final version (Section 2.1). In fact, after the pilot, the main decisions were as follows: to include senior people aged 65 years and over, compared with the initial decision to include only those aged 75 years and over; to consider both cohabitant and hourly (daily/nightly) PCAs; and to consider the absence of support from close family members living in the same urban block/rural building, instead of those living more generally in the same municipality. The interviewers also audio-recorded and transcribed the narratives verbatim, replacing the sensitive information of the respondents (i.e., name, address, and telephone number) with alphanumeric codes (for privacy purposes).

### 2.3. Measures for Mixed-Methods Data Collection

This is a mixed-methods study, with both qualitative and some quantitative data collected in parallel/concurrently [43], but also with a predominant qualitative section/orientation [44].

The topics to be explored, through semi-structured interviews, were generally based on questions adapted from previous authors [45]. This approach allowed for the construction of a preliminary conceptual framework, which was the “theoretically based” structure of the questionnaire/topic guide. This included terms/aspects that are relevant to the phenomenon to be explored, drawing on the existing literature and on the experience of researchers, as key concepts guiding the questions to be proposed. The quantitative/closed-ended questions focused on basic socio-demographic characteristics, physical/functional limitations in daily living activities, and mobility. The qualitative/open questions, which constitute the main part of this study, explored the following aspects: diseases and use of drugs; private and public healthcare services used, including GP; and barriers to accessing and using health services.

Mobility was assessed with a closed question regarding the possibility of moving in the home and outside with help. Regarding the latter, the frequency (number of times per week) was also considered, and physical/functional limitations (self-reported) were assessed with 12 ADLs and IADLs, including 2 specific mobility limitations (going up/down the stairs and bending to pick up an object from the ground) and sensory limitations in hearing and eyesight [16,46]. The ADLs assessed the following abilities: getting into/out of bed, sitting/rising from a chair, dressing/undressing, washing hands and face, bathing or showering, and eating/cutting food. The IADLs assessed the following activities: preparing food, shopping, cleaning the house, washing the laundry, taking medication at the right doses and at the right times, and managing finances. The functional limitations were defined with three levels of difficulty: capacity to perform daily activities autonomously/alone, with help (of persons or aids), or not at all.

Starting from the underlying question guiding the qualitative section of this research, i.e., how older men and women experience frailty and ageing in place alone in Italy with regard to accessing and using health services, some open questions on health and the use of services, considering the year before the interview, were proposed as follows: “With regard to your health, do you suffer from some pathologies that cause pain and require the use of health services?; “If so, could you please describe your experience with them, including the use of some drugs in this regard?”; “Could you please describe your need and use of private and public health services, e.g., GP, MS, and diagnostic tests?”; “Do you encounter some difficulties contacting/obtaining the services, i.e., barriers that hamper/limit both their access and use, e.g., high cost, distance, and long waiting lists?”. It should be clarified that barriers were considered in a general sense, not in terms of each health service individually, in order to understand the issue without causing our older respondents undue stress.

### 2.4. Mixed-Methods Data Analysis

In this study, a mixed-methods design was also used for the analyses (in addition to mixed-methods for data collection, as described above in Section 2.3). It was carried out using the following steps: (1) content analysis of qualitative data [47]; (2) quantitative analysis of certain dimensions collected with closed questions [48]; and (3) quantification of statements for presenting an introductory picture of the phenomenon for synthesis purposes [49]. In particular, content analysis represents a descriptive qualitative approach to data analysis, which is fairly simple but nevertheless generates knowledge, evidences meanings, entails the interpretation of the topic being studied, and allows for the gathering of ramifications and variations in the phenomenon being explored [50]. With regard to data integration and consistency between the quantitative and qualitative analyses, the qualitative accounts from the narratives guided the identification of the main categories, and the quantification of the latter (by gender and with related percentages) allowed a quantitative analysis (without the aim of statistical value) to support the interpretation of the results as a whole [43], with qualitative analysis in turn substantiating and validating the quantification itself. Integration was thus assured at both the interpretation and reporting stages, using narratives, the quantification of statements as data conversion, and “joint display” [51] (p. 2136). The analysis of the 120 interviews was performed by differentiating between males and females. Possible differences across regions and urban/rural areas were not explored, as we aimed to focus on the Italian context and gender dimensions in terms of health conditions and health services utilisation. The steps of the mixed-methods analysis are described in more detail below.

#### 2.4.1. Qualitative Analysis

The standard phases of the framework analysis technique [52] were used to analyse the open answers. This approach involved the following steps: the in-depth and repeated reading of the transcribed interviews in order to gather first impressions and considerations; the identification of macro-/sub-categories, on the basis of the preliminary conceptual framework that guided the editing of the semi-structured questionnaire; the identification of codes drawn from both the concepts used for the interviews (deductive approach) and adding further details that emerged from the collected data (inductive approach); the breakdown of the narratives in charts (two-way matrices, with categories in columns and cases/interviewees in rows), also carried out by refining categories and codes; and the interpretation of the results according to the main similarities and differences that emerged [53,54]. The four co-authors (M.G.M., S.Q., M.S. and G.L.; two PhDs and two MSs; two females and two males) are senior sociologists/gerontologists and health services workers, with extensive expertise in issues regarding older people and formal/informal caregiving, in addition to significant expertise in both qualitative and quantitative research methods. We collaborated on the steps described above. Specifically, M.G.M., S.Q. and M.S. were involved in the identification of macro-/sub-categories and codes; M.G.M. and S.Q. were involved in the building of the charts; and M.G.M. and M.S. carried out the content analysis by measuring the frequency of different categories to identify and interpret both the underlying meanings and relationships among the emerging concepts/patterns [55]. However, all co-authors discussed and contributed to the interpretations of the relevant contents by gender, with constructive consultations when necessary, to address and manage possible disaccords by means of an iterative process. The categories and coding that emerged are presented in Table 1.

The analyses of the 120 interviews were conducted progressively, first identifying the results of single sites (three urban cities and three rural/inner areas) in each region, and then combining the data which emerged. This assessment/management of qualitative results, in addition to the preliminary conceptual framework that was included in the topic guide, allowed manual content analysis, without the use of a dedicated software, in accordance with other studies [56]. Additionally, an in-depth consideration process, which was based on line-by-line readings of the narratives, was used [38].

We did not include hospital admissions as public health services in our study in order to focus on individual services that are also used in hospitals. We also considered that hospital admission often implies, in itself, the use of diverse public health services, and thus, in our opinion, merits a separate dedicated study. Also, in this study, we preferred to use the number of drugs used per day to indicate the intensity of the assumption, instead of using labels such as “few”, “some”, and “many/several” drugs, to simplify the reading of the results, which also has precedent in the literature [57]; in addition, we converted of some contents of the qualitative interview transcripts into numerical category scores, i.e., quantified–qualitative values. This leads to possible hybrid coding in qualitative research, where numerical values can also provide meaningful insights and facilitate comparisons. The relevant verbatim quotations that emerged in the transcribed interviews [58] specified/enriched the qualitative analysis of the findings. Each quotation was coded with “IT” (Italy), a progressive interview number (1–120), and the gender of the respondent, i.e., “F” (female) and “M” (male). Notably, the above quotations are all from women due to the greater number of female respondents and corresponding number of quotes in the sample (90 vs. 30). Also, with the aim of facilitating and simplifying the reading and comprehension of the excerpts from the narratives (sometimes containing dialect terms), the answers were edited to a certain extent; however, full respect to the original meaning was given.

#### 2.4.2. Quantitative Analysis

In addition to the quantification of drugs used per day, the first author, M.G.M., used Microsoft Excel 2024 (Microsoft Corporation, Washington, DC, USA) to provide simple elaborations by gender (percentages of univariate and bivariate analyses) and present these in tables, in terms of the quantitative data collected using closed questions (e.g., socio-demographic, activities performed alone/with help/not able, and mobility). Regarding mobility, the capacity to move only/mainly within the home also included the ability to move outside very rarely, i.e., less than two times a week and only if accompanied or with aids (cane, walker). The capacity to move outside, with or without help, was thus considered as such when it occurred at least two times a week. Regarding functional limitations, four levels were considered, defined using number of “not able” activities reported by senior people: “mild”—when no “not able” activities were reported; “moderate”—when one or two such activities were reported; “high” and “very high” —when, respectively, senior people were not able to perform three, four, five, or more activities [59]. Also, the quantification of statements by gender offers a preliminary synthesis of qualitative results. In this respect, a conversion mixed method analysis was carried out, i.e., the same findings were presented and examined both qualitatively and quantitatively, with a “qualitative to quantitative” procedure [49]. This, however, implies the need to balance “numerical precision with narrative complexity” [60] (p. 208). Both absolute (n) and percentage (%) values are presented in the tables (apart from Table 1, with categories and coding). In some cases, totals do not correspond to 100% when some percentages have been rounded or when a single senior person reported more responses for a single question or, conversely, did not respond. Following the qualitative dominance of this study, quantitative data do not constitute principal results needing statistical evaluation, and the tables do not present the significance level and standard deviation values. More information (on the study design, recruitment and instruments, data collection and analysis) is available in a previous publication [35], on which the present Section 2 is partly based.

#### 2.4.3. Trustworthiness and Rigour of the Qualitative Data Analysis

To ensure the trustworthiness of the qualitative data analysis [61], some fundamental criteria were followed: credibility, dependability/auditability, confirmability, and transferability.

The credibility of results was achieved through the following: the use of a semi-structured questionnaire/topic guide partly based on validated topic guides, which were already administered in previous studies on older people experiencing frailty (e.g., ADL and IADL scales); several peer debriefing meetings among researchers and interviewers (all with long-lasting expertise on the issue of ageing in place and qualitative research) to finalise the protocol, the edit the questionnaire/topic guide, and to define the steps regarding data analysis; and a pilot test allowing the refinement of the preliminary protocol/questionnaire.

The dependability/auditability of the process was achieved through rigorous and accurate documentation/description of the research procedure/protocol (e.g., data collection, transcription of the narratives and analysis of results, and possible refinements of the study design). This functioned as a transparent audit trail, allowing potential external reviewers to check and replicate the entire study procedure. Dependability was also ensured by utilising a sustained collaborative approach, enabling professional interactions between researchers (for the entire duration of this study and beyond, i.e., during publication), with particular regard to data analysis, when addressing disagreements and jointly agreeing on the final findings.

The confirmability of the research results was achieved through detailed field notes of researchers and interviewers on the data collection and analysis process, including decisions and interpretations to justify the choices made during this study. In addition, dissemination seminars with relevant stakeholders and experts allowed the review and validation of preliminary findings.

The transferability (analytic rather than statistical) of results/procedures to other settings was achieved through the following steps: an in-depth preliminary literature review to analyse previous background results emerging from studies on the topic (e.g., several ISTAT data presented in Section 1); the use of these data as background to provide a starting conceptual framework of the phenomenon; and a comprehensive description of the setting, sampling, and recruitment process of participants.

With regard to inter-coder reliability, three researchers (M.G.M., S.Q. and M.S.) coded the transcripts (40 each); compared the results; solved disagreements (i.e., how the same data should be coded); and reviewed the codes when necessary, with the help of G.L. [62].

The “COnsolidated criteria for REporting Qualitative Research” (COREQ) (Supplementary Material, File S1) were used as a checklist to help report this study and ensure rigour [63].

## 3. Results

### 3.1. Main Sample Characteristics

In the whole sample, the respondents were mostly 80 years and over, widowed, with low/medium educational level, and lived largely alone without the support of a PCA. A slight majority had a mild/moderate level of physical/functional limitations, and their mobility allowed them to leave their homes with help. Compared with men, women were older (higher proportion in the 80 years and over age group) and were also predominantly widows and living alone. On the other hand, men were more prevalent among those with higher levels of functional limitations and mobility only/mainly in the home. Both males and females reported a high level of primary/middle school education (Table 2).

### 3.2. A Preliminary Quantification of Statements by Gender

#### 3.2.1. Main Diseases and Use of Drugs

**Figure 2:**
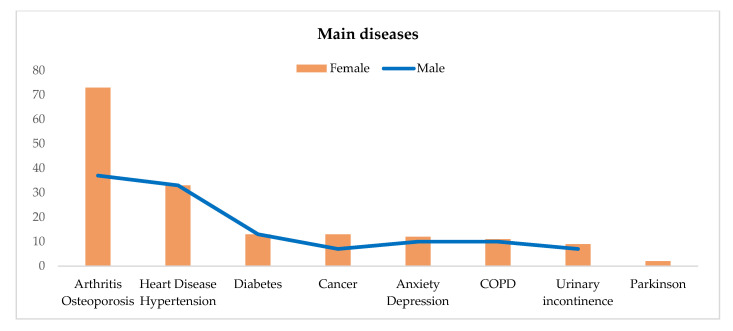
Main diseases by gender.

With regard to the main diseases, senior people reported arthritis/osteoporosis (64%) and heart disease/hypertension (33%), in addition to diabetes (13%), cancer and anxiety/depression (12% both), and COPD (11%). Compared with men, older women suffer more from arthritis/osteoporosis (73% vs. 37%) and (slightly more) from cancer, anxiety/depression, COPD, and urinary incontinence. Parkinson’s disease was reported by only two women. Older people also use many drugs, with a greater intake among women (Table 3; Figure 2).

#### 3.2.2. Main Public and Private Health Services Used

Apart from help from their GPs, which was reported by all the respondents, slightly more than half of the sample (both genders) used health services, especially those delivered by the public sector (59% vs. 55% in total). However, women used services in both sectors in a balanced manner (59–58%), whereas men used more services from the public sector (60% vs. 47%). Also, only 29% used both public and private health services, and these were mostly women (32% vs. 20%) (Table 4).

**Figure 3:**
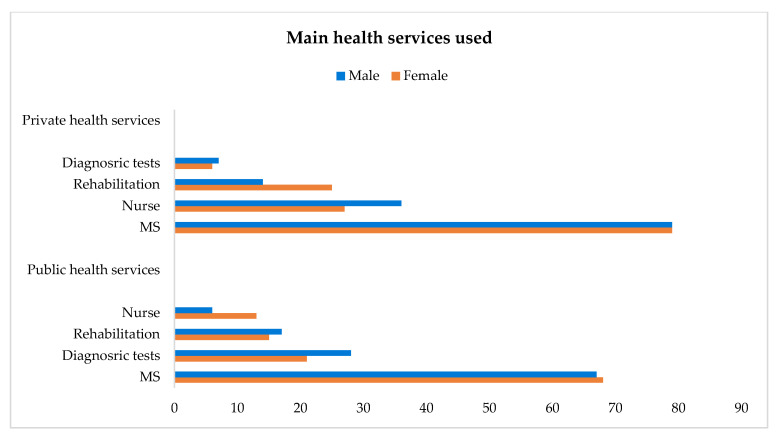
Main public (apart from GP) and private health services used by gender.

When moving from the general use of public vs. private healthcare to single types of services in each sector, the gender dimension takes on more nuances/declinations, as a result of more types being reported by each respondent in some cases. With respect to public health services, senior people reported more than one in some cases; in particular, MSs (68% in total, and similar for both genders) and diagnostic tests (23% in total, more for men), followed by rehabilitation (15% in total, slightly more for men) and nursing care (the latter reported predominantly by women). Some health services are, however, more requested from the private sector, especially MSs (79%, both genders), nursing care (29%, more for men), and rehabilitation (23%, more for women) (Table 5; Figure 3).

#### 3.2.3. Main Barriers Preventing the Use of Health Services

**Figure 4:**
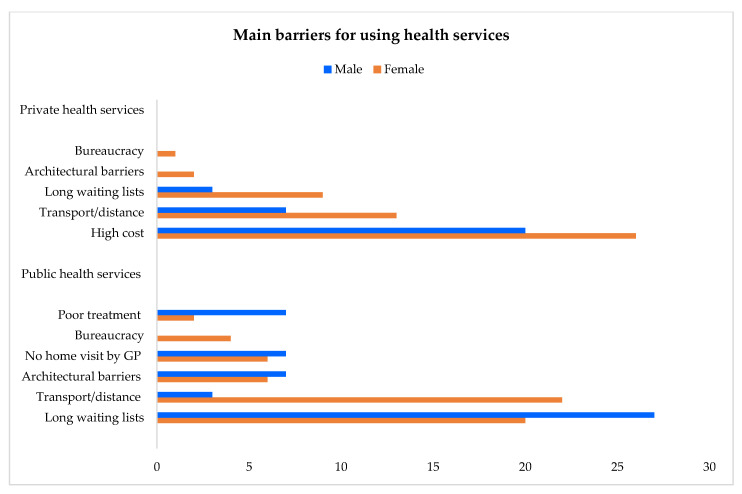
Main barriers for using public (including GP) and private health services by gender.

The greatest barriers to using public health services are the long waiting lists (22%) and the need for accompaniment/transport to reach a necessary destination, which is often very distant from an individual’s home (18%). These two barriers are more commonly reported on by men (27%) and by women (22%), respectively. Other obstacles reported by both genders are architectural barriers and a lack of availability to provide home visits by GP. Bureaucracy was complained about only by women, and poor treatment was brought up by men especially. In the private health sector, major barriers are the cost of services (24%, more for women), the need for accompaniment/transport, and, in some cases, long waiting lists (12% and 8%, respectively, more for women) (Table 6; Figure 4).

### 3.3. Original Words from the Narratives by Gender

#### 3.3.1. Diseases and Drugs

The narratives highlight well how multiple pathologies, particularly arthritis/osteoporosis, are very common in older individuals, especially women. However, heart disease/hypertension and diabetes also represent serious illnesses for both genders. These pathologies negatively impact the autonomy of senior people.

*My health is chronically bad! I have arthrosis, hypertension, and a herniated disc. I have great difficulties in walking, I fear falling. (IT-112_F)*

*I have advanced diabetes and physical stability problems. I have a lot of illnesses! (IT-97_F)*

*I have severe osteoarthritis. I cannot carry weights. I cannot do physical efforts because I have* *pain in both arms and legs. If I walk for a long time, I get short of breath.* *(IT-58_F)*

*I recently had a heart attack and this has greatly compromised my health and reduced my mobility capacity. Furthermore, I am diabetic. (IT-104_M)*

Women reported other illnesses slightly more than men, e.g., cancer, anxiety/depression, COPD, and urinary incontinence.

*I have asthma due to air pollution. Also, some years ago I had a breast cancer. (IT-95_F)*

*I had several fractures and I also suffer from depression following the death of my husband. I have been treated by several psychologists but I have not yet resolved it. (IT-93_F)*

*I have Parkinson’s disease and also high blood pressure. (IT-16_F)*

*I had several surgical interventions. Five years ago I broke my femur. In addition, I suffer from urinary incontinence. All this leads me to be depressed! (IT-45_F)*

Also, polypharmacy frequently occurs in later life, with senior people using many drugs, especially women.

*I am stuffed with everything, every type of drug, even morphine to ease the pain. (IT-21_F)*

*I take drugs for the heart, for blood pressure, for cholesterol. I take 14 drugs in a day! (IT-7_F)*

*I can still manage to take the medicines on my own since they are still few, four pills a day, for blood pressure and the prostate. (IT-89_M)*

#### 3.3.2. Use of Public Health Services

Senior people need more than one type of public health service, and MSs are commonly used by both genders. However, the topic of public health services highlights the role of the GP in Italy, who is used by all respondents, both males and females, since the formal prescription by this health professional is necessary for senior people to benefit from free medical visits and exams in the public sector.

*I need the visit of several MSs, and I need the GP’s prescription to avoid paying. (IT-105_F)*

*Over the past year, I had a visit by two MSs. I booked the visits through the National Health Service with the GP’s prescription. I paid nothing. (IT-20_M)*

Also, diagnostic tests (above all in hospital) and rehabilitation are used largely by older men, and nursing care mainly by older women.

*Every month I use day hospital for several diagnostic tests, in particular blood test. (IT-14_M)*

*For physiotherapy my GP prescribed a 60-day treatment. I am waiting that the public physiotherapist comes to my home. (IT-91_M)*

*I need a public nurse, approximately three times a week, for wound dressings. (IT-27_F)*

#### 3.3.3. Use of Private Health Services

Senior people, both genders, turn to private health services, especially MSs, followed by nursing care, mainly men, and rehabilitation, mainly women.

*I see the cardiologist to carry out checks every three-four months for a cost of a few hundred EUR per visit. This MS visits me at home. Another private service I use is the nurse who takes my blood at home for the test. For this I pay 10 EUR to the private nurse. (IT-104_M)*

*I go regularly to a couple of private MSs or they come to my home. I am treated by a cardiologist and a gerontologist. I see them five-six times a year. I have also a therapist who comes to my home twice a week and I pay 50 EUR a week. (IT-92_ F)*

#### 3.3.4. Common Barriers to Public and Private Health Sectors

- **Long Waiting Lists**

Long waiting lists represent significant obstacles for using public health services (more for men), and they are also sometimes reported in the private sector (more by women). The paradox is that some senior people prefer to pay for private services (when they can financially) to avoid waiting too long.

*I booked a visit with a urologist in the public healthcare sector and I had to wait about one year before being summoned! (IT-96_M)*

*I prefer the private sector. When you pay, the waiting list is less long! (IT-12_F)*

In particular, senior people in the public sector have to wait several months before receiving physiotherapy treatment.

*To benefit from a cycle of massages by the public physiotherapist, I had to wait a lot of time, but I cannot always wait so much. The problem is that in the private sector I have to pay 30 EUR each time! (IT-9_F)*

- **Need for Accompaniment and Transport**

The need for accompaniment/transport to go where services are supplied represents another significant barrier for both public and private healthcare sector access, especially for women.

*Public health services, especially MSs, are far away. There is no one who can accompany me! This is a great problem. (IT-112_F)*

*I need to leave my region to have a private visit by an MS, and each time I have to pay the cost of both the transport and the visit. (MAR_34 F)*

In particular, public transport does not seem adequate for older people. When possible, children can be a support in this regard.

*An older person who wants to get a bus risks a lot! Bus stops are very poor, and are often not clearly indicated. (IT-87_F)*

*When I need a medical visit, I can trust my children, this is a great fortune! (IT-118_F)*

*The rare times I go to the GP, the PCA has to accompany me, I cannot go alone. (IT-90_M)*

- **Architectural Barriers**

These structural barriers are reported by both genders and highlight how healthcare buildings are not always easy to access, e.g., when laboratories are located on the upper floors, with multiple flights of stairs and elevators that do not work.

*I use a wheelchair, with several problems when there are stairs and no elevator! (IT-95_F)*

*The building where my GP has his medical office does not have adequate access for disabled people, thus I need the help of his secretary to reach the GP. (IT-91_M)*

- **Excessive Bureaucratic/Administrative Practices**

A problematic excess regarding this factor was reported only by women in both the public and private sectors. This context can, for example, result in waiting several months to receive necessary documentation, e.g., to benefit from a public walker.

*When a senior needs a particular health service, e.g., the supply of diapers, a lot of formal documents need to be filled in! (IT-54_F)*

*To have a public walker, a relative of mine had to make several trips between GP and other offices, and several documents have been requested! (IT-45_F)*

#### 3.3.5. Barriers Reported Only in the Public Sector

- **No Home Visits by a GP**

Minor barriers were reported by both genders, e.g., no availability for GPs to provide home visits.

*GP does not make home visits anymore! He does not even know where I live. (IT-36_F)*

*I rarely see my GP. It has been a long time since he came to visit me at home. (IT-90_M)*

- **Poor Treatment**

Poor treatment by public health services has been reported, especially by men. Sometimes this is due to several hours of waiting before being accepted by an emergency room or to “insensitive” health professionals.

*Last year I went to the emergency room and I waited five hours in a wheelchair. I am old and I suffered a lot! (IT-104_M)*

*Last summer I called a medical guard at night, who answered that she had no obligation to come home! Thus, I called the police. (IT-91_M)*

With respect to GPs, poor treatment was described by older women as a limited propensity to allow physical visits to the medical office. Consequently, only/mainly prescriptions (of drugs, diagnostic tests, and specialist visits) and the “delegation” of medical services in case of health emergencies are provided.

*GP never visits me in his office. He only writes prescriptions. I turn to MSs. (IT-99_F)*

*When I have some urgency my GP tells me to call the emergency room. (IT-16_F)*

#### 3.3.6. Barriers Reported Only in the Private Sector

The high cost of private health services has been highlighted above all by women, especially for MS visits.

*Currently private MS visits are not affordable for me. I have a little pension that does not allow me to benefit from general private health services. (IT-65_F)*

*If I pay a private healthcare visit, then I have no money for daily needs. (IT-100_F)*

*I spent a lot of money on visits by MSs! Some took 100 EUR, and some took much more. (IT-99_F)*

*I am forced not to use private services because of their excessive cost. (IT-66_M)*

## 4. Discussion

This study focused on older people experiencing frailty and ageing in place alone in Italy and aimed to analyse the gender dimension with regard to health status, the use of drugs, and barriers to accessing health services. The findings indicate that women reported more major pathologies than men, especially arthritis/osteoporosis. Also, even though, in general, males and females use almost the same services and face the same obstacles in terms of access, some differences in this respect emerged, as discussed more thoroughly below.

### 4.1. Multimorbidity and Polypharmacy: The Male/Female Dimension

Multimorbidity and polypharmacy are common in both older men and women. Our study revealed several pathologies, mostly arthritis/osteoporosis and heart disease/hypertension, followed by diabetes. However, more cases of multiple diseases, especially arthritis/osteoporosis, were registered among females, and Parkinson’s disease was reported only by two women. Furthermore, women used more drugs than men. The picture above is generally confirmed by the literature for Europe [9], stressing how the greater prevalence of age-related conditions in women is also associated with their longer life expectancy. Women indeed live longer than men and thus have more years potentially affected by disability, which in turn reduces the gender gap in terms of the number of years spent healthy, even though 37% of older women and 43% of older men report being in good health [19]. Overall, both the national and international literature highlight that women have a more positive life expectancy but also have worse indicators regarding quality of life and wellbeing, and this gender gap cannot depend only on genetic differences. Rather, it is also affected by societal and cultural issues [64].

With regard to Italy, a systematic review of results from the Global Burden of Disease Study in 2021 highlighted gender disparities in both the health status and life expectancy of the population [65]. Additionally, the most common pathologies reported by the ISTAT for senior people aged 65 years and over of both genders [16] are osteoarthritis (48%), hypertension (47%), heart disease (19%), and diabetes (17%). However, older women reported worse health indicators than men, e.g., more severe motor, sensory (e.g., eyesight and hearing), and cognitive limitations (33% vs. 23% on the whole). Older women also showed greater mobility limitations due to the presence of diseases (39% vs. 22%) and greater anxiety/depression and dementia (including Alzheimer’s disease), with rates of 19% and 5% vs. 9% and 5%, respectively. OECD data for Italy [25], in particular, report that women suffer from depression more than men (7% vs. 3.5%) and make greater use of antidepressants. Concerning women in Italy aged 75 years and over, further studies [8] revealed that 48% have three or more chronic diseases, compared to 34% of men. Moreover, 18% of men and 25% of women in this age group reported serious limitations in daily activities due to health problems. Some international studies also support these findings for Europe, and suggest that poor hearing and poor eyesight, which are more frequent in older women [66], amplify their possible negative health outcomes [67]. Another study in Germany reported that about 32% of older people were treated for five or more chronic diseases, with a mean number per person of around four diseases for women and three for men. According to other authors, hip and knee osteoarthritis are particularly relevant to women’s health [68], and the prevalence of joint and bone diseases is much higher for women (52%) than for men (36%) [69]. In particular, hip fractures are frequent for senior people with skeletal frailty due to osteoporosis, especially older women, who indeed suffer from both arthritis and osteoporosis more often than men [19].

It is worth considering that age and related physiological changes and reduced physical activity are important variables for the vulnerability of older people, with those aged over 80 years being more exposed to chronic diseases and mobility issues [9], and this in turn also leads to the use of multiple medical treatments to address health problems resulting from multimorbidity. In particular, regarding the use of drugs, a recent study in European countries [70] reported that, generally, the prevalence of polypharmacy (measured as at least five different drugs taken on a typical day) increases with age, from 30% for those aged 65–74 years to approximately 50% for those aged 85 years and over, with older men reporting a polypharmacy prevalence lower than women in most countries. Another source for Italy [71] similarly highlights how the use of four or more different drugs increases with age, from 28% among those aged 64–74 years to 45% among those aged 74–84 years and 59% among those aged over 85 years, even though significant differences by gender did not emerge (40% of females and 39% of males use four different drugs). Conversely, a further study on the prevalence of polypharmacy (measured as having five–nine drugs prescribed during a six-month period) in older Europeans [72], revealed a value of 58% for females and a lower 42% for males in Italy. Also, according to a survey by ISTAT [30], in Italy, older women used drugs more than older men (in the four weeks preceding the interview) prescribed by GPs and/or MSs (67% vs. 62%). Another study carried out in southern Italy in 2018 [73] also reported a greater prevalence of drug use among females than males aged 65–84 years and over, and related prescriptions (per 1000 treated patients per day) were especially higher among women aged 75–84 years (96% vs. 93%).

It is to consider that, in our study, older women reported an overall worse health status than men in terms of their prevalence of reported diseases, although the latter reported higher levels of functional limitations and a lower mobility (only/mainly in the home) than the former did (see Table 2). Conversely, the literature usually reports that older women are more dependent in daily activities than men [16]. The different results in our study could be associated with our particular purposive sample, which includes only senior people with intermediate mobility, living alone or with a PCA, and with no help received by close relatives. Using these characteristics, our sample comprised specific typologies of senior people, e.g., those supported by a PCA, who are usually older men with severe physical limitations [74]. Our specific sample and strict criteria could also be responsible for some of the differences between our findings and those of previous studies on the same topic, as addressed more thoroughly below. However, some studies highlight how the social/cultural construction of manhood could have a role in preventing men from reporting poor health statuses with several diseases, since this could be considered unacceptable masculine behaviour [75]. Also, the higher level of functional limitations of men in our study (which were calculated only with regard to activities that senior people were unable to perform by themselves) could be linked to the fact that, as also stressed by the literature [76,77], household tasks, e.g., cleaning, cooking, and laundry, still remain traditional female tasks, especially in old age, whereas men are more involved in “masculine” tasks, e.g., home and garden maintenance.

### 4.2. Use of Public and Private Health Services by Gender

In our study, senior people used both public and private health services (more the former). In the public sector, the GP or family doctor is used by all, both genders. In Italy, this healthcare professional represents the main reference/contact point for health (prevention, early diagnosis, and treatments) for the entire population, and it is formally necessary to obtain a prescription by a GP for senior people to benefit from free medical visits and diagnostic tests in the public sector (as already discussed in the Introduction) [19,31]. This finding is supported by ISTAT data for Italy [16,30], indicating that (in the four weeks preceding the interview) almost all older people had turned to their family doctor at least once, 91% women and 89% men. EUROSTAT data [34] for the EU-27 also report that 92.3% of older women and 91.5% of older men consulted a GP in the 12 months preceding a survey in 2017.

However, apart from GPs, our findings indicate that, while women used services from both sectors similarly, men especially used those delivered by the public sector. This result could be attributed to the fact that, as reported in the previous literature [9,78], men and women seek medical care from a doctor in different ways, with the former generally being quite reluctant, whereas the latter are generally more careful about their wellbeing and are more willing to use health services. Men prefer to use more user-friendly and comfortable health services, and the public sector, as a “comfort zone”, as mediated by the GP, could be more acceptable/suitable for them, instead of taking initiative and seeking, for instance, proper private health services. This is particularly the case when men are older and widowed—which was the case with most of our respondents—since widowhood in later life is often associated with self-neglect, especially for men suffering the loss of a spouse/partner who could stress/support their health behaviour [79]. In this respect, Comolli et al. [80] found a greater negative effect of widowhood on men than women in Italy from 2014 to 2022, with an estimated increase in the mortality risk, among the surviving spouses, being, respectively, 35% vs. 24% after 67 years of age.

Regarding single types of health services, of those used by each gender, further similarities and differences emerged in our results. MSs, both public and private, are greatly used by both men and women in similar numbers, with specialists from the latter sector being prevalent. With regard to other health services, some mixed results were found in our study, with prevalent use by males or females depending on the type and sector. Diagnostic tests, especially public ones, were more commonly used by men. Rehabilitation was (slightly) more reported by men for the public sector, and more by women for the private one. Nursing care was reported more often by women for the public sector, and by men for the private one. These findings are almost/partly supported for Italy in previous ISTAT data [16,30], which are, however, less detailed in terms of sector. They suggest that about two out of three older people have resorted to specialist visits, i.e., 67% of women and 66% of men, and about 40% were free for both. Also, 48% of older males and 50% of older females used diagnostic and laboratory exams, and around 52% were free for both. The same sources reported that 11% of older men and 15% of older women used rehabilitation physiotherapy. Some small discrepancies between our results and the ISTAT data could be attributed to the different periods considered for the survey, that is, only the four weeks preceding the interview were considered for ISTAT, whereas the previous year before the interview was used in our study. Also, our strict criteria (as outlined above in Section 4.1) include only certain typologies, i.e., senior people with functional limitations and at least an intermediate level of mobility, whereas ISTAT considers individuals who are residents in Italy (in 2019, same year of our study), aged 15 years and over, and generally includes senior people aged 65 years and over without a preliminary selection on the basis of health/functional conditions [30]. Other authors, however [78], support our results regarding the greater use of diagnostic tests by men, suggesting that “men have a very functional view of their bodies and see healthcare as a ‘fix-it’ cure; therefore, they may respond better to healthcare interventions that offer tests, facts and figures, e.g., cholesterol, blood pressure” [78] (p. 2).

Further considerations can be provided with regard to the (slightly) greater use of public sector rehabilitation by men and private sector rehabilitation by women, as observed in our study. It can be assumed that women, as a result of having higher levels arthritis and osteoporosis than men, have a more urgent need for private rehabilitation at home (when financially affordable for them), which is a service very difficult to obtain in Italy in the public sector. This is due to very long waiting lists [31,81], as also reported in our study by a woman who had to wait several months before receiving public physiotherapy treatment. Rehabilitation in Italy is also delivered in low-intensity public care hospitals, but these have an insufficient number of available beds [31]. Data from the Italian Ministry of Health [82] more precisely report that in 2023 (with regard to the whole population, but particularly for those aged 65 years and over), therapeutic rehabilitation institutes/centres (with residential, semi-residential, and home-based interventions) were predominantly accredited private structures (943 units, with about 25,000 residential and semi-residential beds), with fewer public ones (260 units, with about 2500 beds). Moreover, motor/functional rehabilitation recorded about 33,000 residential and semi-residential users, whereas about 1100 and 20,000 need cardiac and neurological rehabilitation, respectively. This source [82] does not provide specific data on rehabilitation at home by the gender of users, but reports the number of hours of public therapy within integrated professional home care, and in this respect, only three hours (and four accesses) are indicated for each treated senior person in 2023.

Concerning the use of professional nursing care, as emerged from our findings, it could be possible that women resort more to the public sector, for instance, when they need injections, which are easier services (as for timing) to obtain at home in Italy. Also, the fact that our male respondents resort more to private nursing care could be attributed to their greater use of diagnostic tests, which can also involve private and more comfortable blood drawing at home (instead of in a medical unit/laboratory). INPS data [83] indicate the better financial situation of older men than older women, with an annual income/pension in Italy that is indeed higher for the former by about 35% (around 24,700 EUR vs. 18,300 EUR in 2023), due to different patterns in labour market participation throughout life course. This could have a role in directing men towards the private sector when they need a service more frequently, such as nursing care.

### 4.3. Barriers for Using Public and Private Health Services by Gender

In Section 4.2 above, we partly/indirectly outlined the issue of barriers limiting or even preventing the use of public and private health services (i.e., long waiting lists and cost), with hypotheses related to some particular cases/types of services. These barriers are discussed below in more depth and are further analysed by sector and gender.

In the public health sector, the main barriers reported by our respondents are the long waiting lists, slightly more for men, and the travel distances and consequent need for accompaniment/transport, almost exclusively for women. In the private sector, largely for women, the main obstacle is the cost of services, followed by waiting lists and travel distance. Barriers to using health services are thus a predominantly female issue, with older women even forced to give up medical care or examinations/treatments if these are too expensive (especially for private MS visits), if the appointment is scheduled too late compared with the need, or if they cannot reach the place where the service is delivered by themselves, and taking a bus by themselves is difficult.

The above findings first indicate that all these barriers reflect the burden of disease on the participants with functional limitations in our study, but poor economic situations, especially for women, and a limited support network, are also relevant. In particular, low income may affect the availability of a car, which can be an issue when a healthcare service is distantly located. In addition, there is often a lack of available relatives who could accompany the individual and public transport is often not adequate for older people experiencing frailty, especially in rural locations, which can lead to renouncement/delays in treatment and, consequently, to possible disease complications. Similarly, short waiting times, especially for public health services, are crucial for senior people with worse health conditions, but similarly, economic issues and a lack of relatives who can financially support them can limit the possibility of paying for faster, but more expensive, private visits/treatments.

More generally, and aside from gender differences, several sources reported long waiting times, financial problems, and geographical barriers or long distances to reach the healthcare facilities, in addition to a lack of transport services, as barriers to using health services in Europe [19,84]. A study on the factors that influence the unmet healthcare needs of older people living in 28 European countries in 2019 [85] found that greater problems were observed among those with lower income and severe functional limitations, with high costs, too-long waiting lists, and distance or transportation issues emerging as the leading obstacles. This highlights that patients in the EU-27 often have to contribute their own money (cost-sharing) to benefit from public health services/outpatient care. These out-of-pocket payments in 2022 were high, especially in Portugal (52%), Italy (47%), and Ireland (40%) [19]. In particular, OECD data [25] indicate that, in 2022, 3.3% of Italians who were in the lowest income quintile, and less than 1% of those who were in the highest one, reported unmet health needs. Notably, in recent years, the incidence of poverty among older people in Italy has decreased, with greater spending capacity of the oldest adults resulting from higher average incomes and savings accumulated throughout life [86], even though the degree of disability and healthcare costs represent significant factors impacting the risk of poverty among dependent senior people [87]. In particular, 50% of older people living alone in Italy do not exceed the threshold of 16,879 EUR per year (1406 EUR per month) [88]. Kalavrezou et al. [9] also reported that in 2019, 10% of the EU-27 population aged 65 years and over with functional limitations were not able to access healthcare for economic reasons, and in Italy this rate was higher (about 14%).

With respect to gender differences, several sources highlight, as in our study, a gender gap regarding reasons leading to delayed/not performed health services for senior people.

For Europe, EIGE [89] indicates first higher costs for females (40% vs. 34% of males), followed by longer waiting lists for males (25% vs. 23% of females), as the main obstacles leading to unmet health needs among older people aged 65 and over. In addition, older women with functional limitations experience obstacles in accessing appropriate transport to reach healthcare providers. The OECD [19] highlights that in the EU-27, older females especially have fewer financial resources for health services, which leads to unaffordable costs and unmet medical care needs. Moreover, men are more likely than women to delay medical visits or examinations (19% vs. 14%), “in the hope that the health issue will resolve itself” [89] (p. 106), which is in line with the lower male propensity to seek medical care from a doctor (as indicated above in Section 4.2).

Similarly, in Italy, for the two-year 2022–2023 period [71], the National Institute of Health reported that 25% of older women and 21% of older men gave up at least one medical visit or diagnostic tests that they would have needed in the 12 months preceding the interview. This is mostly due to long waiting lists, difficult access to the facility (location too far away, lack of transportation, inconvenient hours), and economic difficulties. Previous ISTAT data for Italy [16,30] also highlighted senior people who had given up on treatments (in the four weeks preceding the interview) due to economic reasons (9% for men and 12% for women), long waiting lists (19% vs. 20%), and problems with transport (7% vs. 8%). This last obstacle is, however, more pronounced/evident, and is the main issue for women in our study.

De Belvis et al. [31] confirmed that, in Italy, the main reasons making people (both genders) dissatisfied are excessive waiting times (44%) and costs (24%). They also added that patients who choose to pay for a private health service have more choice, for instance regarding MSs, and the waiting times are much shorter, e.g., 6 days instead of 56 days for a private orthopaedic consultation vs. an equivalent NHS service. However, our results revealed that, in some case, long waiting time were still extant also in private health services, especially for women. This represents quite a paradox, since older people turn to the private sector to avoid the excessive waiting lists affecting the public sector. In this regard, Fjær et al. [90] found that patients with greater financial resources in particular can generally overcome waiting lists with less trouble. However, in some cases, senior people with lower incomes could be forced to use their own savings or take on debt to cover expenses for private health services, which are generally spent on MS visits [31]. Waiting times in the private sector could thus be a reflection of the greater switch from public to private healthcare in last years in Italy, with the consequence that a greater demand in the latter sector, in turn, increases the related delivery times. Private health spending has indeed increased in recent years in Italy, despite a low increase in the income of families, due to a weak economy [31]. This “loop” is crucial for older patients with multimorbidity and chronic diseases, since delayed or missed appointments and difficulties accessing healthcare services could imply unmet urgent health needs and poor health outcomes [84]. Privatisation is occurring throughout the Italian NHS, which is thus developing into a private sector, and the issue of (un)equal access of citizens to healthcare services has also emerged [65].

Further minor barriers were reported in our study by both genders, namely architectural barriers and a lack of home visits by GPs and in the public sector, whereas complex bureaucracy was discussed only by women and poor treatment in the public sector was especially discussed by men.

Various authors stress these issues in Italy [91,92] and suggest, in particular, how structural obstacles in buildings, especially the presence of multiple flights of stairs and the absence of handrails or elevators can discourage senior people from moving, both physically and psychologically. Other authors [93] highlight that, in general, GPs who avoid home visits and have contact with patients only by telephone or through relatives or who suggests resorting to the emergency department for urgent needs cause senior people significant suffering. Conversely, such visits can strengthen relationships between GPs and patients, especially for senior people with multimorbidity experiencing frailty. It is, however, worth considering that in recent years, in Italy, the number of patients assisted by GP has greatly increased (over by 1500 since 2018), which has resulted in the reduced availability of time that can be to be devoted to home visits [94]. Also, according to the OECD [19], in 2022, there was a shortage of GPs in several EU-27 countries (75%), including Italy. In particular, the percentages of GPs and MSs were, respectively, 21% vs. 68% in the EU-27 and 16% vs. 81% in Italy. Other doctors were 11% in the EU-27 and 3% in Italy.

Other studies have stressed how the public health sector is particularly affected by organisations and bureaucratic/administrative procedures, which are cumbersome and negatively influence the supply of services [95,96]. This context could affect older women more, as, for instance, the process of an individual attempting to obtain an aid could involve going to various administrative and public offices. Thus, the problems of arthritis/osteoporosis limiting movement and the need for transport resurface for these individuals. Some studies also address the issue of poor treatment and outline concerns regarding the shortage of available staff in the face of multiple patients, especially older persons, and in periods with the highest numbers, this leads to several hours of waiting for treatments and limited attention by nurses and physicians [97]. This complaint in our study is reported more by men, and this could to some extent be related to the fact that, as evidenced by the literature [98], women are generally more resilient than men and are also more adaptable to adverse circumstances, despite social norms placing men in a strong male role. In this regard, Gordon and Hubbard [99] suggest that certain social and even biological aspects play a role in such a picture, and resilience and wellbeing, especially in older men, decreases when they lack the support and love of a spouse/partner and live alone, as is the case in our study [100].

Interestingly, a recent source for Italy in 2024 [81] reported findings regarding the factors that lead to unmet healthcare needs, which are similar to some of our results, but include more detail. In general, around 47% and 50% of senior people complain, respectively, about long waiting lists and difficulties in accessing health services (e.g., when booking a medical visit) and obtaining clinical information and documentation. In particular, outpatient and home rehabilitation care, in addition to long waiting lists, are not used due to the following reasons: the general difficulty in access (20%); a lack of provisions for a certain type of rehabilitation by the NHS (18%); suspensions/reductions in the service (15%); the lack of guidance regarding access methods (11%); and the poor quality of the service (10%). These data confirm how rehabilitation care provided by the NHS is not sufficient to meet the needs of patients, and this shortcoming is replaced by booking and paying for private rehabilitation (as already stated above in Section 4.2).

Notably, in our study, barriers are in some cases reported even by those who do not use the services at all. This reinforces the crucial issue of obstacles not only limiting but also preventing the use of health services by older people experiencing frailty, with functional limitations and living alone, especially females.

### 4.4. Implications for Practices and Policies

The recent rapid ageing of the population, with increasing functional limitations and complex health needs among older people, related to a decrease in autonomy, has led to an increase in healthcare consumption, especially MSs, both in the public and private sectors. This could have some implications for policymakers in terms of care for both genders, with particular attention given to older women. However, to reduce gender disparities, “the specific health needs of males and females must be addressed through targeted interventions” [65] (p. e318).

The first general consideration involves both multimorbidity and polypharmacy, especially for older women. This requires even more a multidimensional and comprehensive geriatric evaluation/assessment to detect especially mobility and sensory limitations, in addition to memory problems, with the support of a multidisciplinary care team of professionals [99] and coordination among GPs and MSs. The “WHO Integrated Care for Older People (ICOPE) Guidelines” recommend the overall assessment and management of the physical and mental capacity of older people by means of community-level interventions in order to promote their wellbeing with regard to their capacities/abilities [101]. This, in turn, requires the necessity of implementing more integrated and less fragmented social care and healthcare provisions to assure the continuity of cure and care in multimorbid older people experiencing frailty across different settings, providers, and levels of care [22]. Currently, in Italy, health and social services are not fully integrated, since they receive different types of governance and funding. A recent national law (Delegated Law n. 33/2023, and its Implementing Decree n. 29/2024) aimed to reformulate the entire LTC system, with guidelines for national coordination among services, for better social–health and formal–informal integration, with particular regard to home services and appropriate funding mechanisms [102,103]. In this context, health professionals/LTC workers, family/informal caregivers, and stakeholders could collaborate and build care partnerships, such as coordination, integration, and mutual recognition for providing care activities, with a vision of integrated LTC, and strengthen the mental wellbeing of everyone [104]. In addition, attention should be paid to the gender dimensions of older people experiencing frailty to develop good practices/interventions that are person-centred and age/gender-oriented. To achieve this aim, medical guidelines and gender-specific protocols could be helpful [105]. The above also supports the consideration that currently, especially in economically developed countries and regions, although life expectancy has generally increased, with higher levels of multimorbidity especially for women, under certain appropriate policy interventions, senior people can maintain both health and longevity, thus contrasting an increasing vulnerability linked also to an extended lifespan.

The main barriers to using health services, which are more evident for females, i.e., long public waiting lists, high costs of private services, and health services not close to where older people experiencing frailty live, require particular attention.

There is an urgent need to reduce waiting times, especially for public rehabilitation at home, which is widely used in the expensive private sector by older women who suffer more from arthritis/osteoporosis. With respect to the problem of long waiting lists and criteria for prioritising services [106], the Italian government has recently adopted the “New National Plan for the Management of Waiting Lists 2025–2027” (an update of the previous plan 2019–2021), in addition to a dedicated national observatory close to regional units [107]. The main aim of the mentioned plan is indeed to reduce waiting times for healthcare services, by means of a monitoring system that allows citizens to receive updated information (via the websites of the Regions and Local Health Authorities) on average waiting times and the availability of health facilities. If the service is not guaranteed in the public sector within the maximum waiting times (according to the indicated priority class), the Local Health Authority to which the patient belongs activates protection paths as alternative ways of accessing services (e.g., private health services reimbursed by the NHS, appointments at unusual times and days, and agreements with accredited private structures). This is also in light of the hard consequences of the COVID-19 pandemic (which occurred after our study, carried out in 2019), which caused the delay/suspension of several health activities, with a consequent increase in waiting times, especially for non-urgent health needs [31,108].

Another important factor is the necessity to provide private health services at a low cost for senior experiencing frailty or to provide them with economic benefits, with particular attention to the lower financial assets of women. Despite this being a well-known gender gap, it is important to further stress the seriousness of this aspect, that also results in greater material and emotional insecurity, especially for women. However, the issue of out-of-pocket payments also needs to be addressed. The coverage policy and financial protection for improving and increasing access to healthcare requires more limited co-payments in the public sector and higher exemptions for frequent users with lower incomes, thus contrasting socio-economic inequalities, in addition to more aligned financial incentives for various providers [19].

The issue of travel distance to health facilities, especially for women, could be addressed by implementing adequate/dedicated public transport services for senior people, e.g., organised and delivered by municipalities. Additionally, help from volunteering associations in this regard could be supported more. Furthermore, a network of “community houses” providing basic/essential health services throughout the national territory, with available GPs, nurses, MSs, and other health professionals, as described in the Italian National Recovery and Resilience Plan (NRRP) [109], could in particular help in managing the problem of geographical distance for using health services, which in turn could improve the wellbeing of senior people [110]. The NRRP also includes actions to manage proper health workforce development in light of the shortcomings of GPs in Italy, including the fact that these professionals are the oldest in the EU-27, raising concerns about their substitution when retired [25].

Further barriers include limited home visits by GPs and architectural barriers limiting access to medical structures/laboratories (for both genders), in addition to poor treatment (mostly for males) and excessive bureaucracy (only for women). Good support by the GP is necessary, with visits at home or at least online (this however requires e-health literacy of senior people and families), as this can reduce the need for emergency departments and hospitalisation [111]. Also, a trusting senior person–GP relationship can strengthen the medical adherence (or compliance) of the former, i.e., the degree to which a patient adheres to medical prescriptions [112]. This approach could improve many of the health outcomes of senior people experiencing frailty; however, it should be managed in light of the increased number of patients attended to by GPs, as already discussed above (Section 4.3). Poor treatment, especially when it occurs in emergency medical units, could be managed by reinforcing available health staff, which is currently in critical shortage in several European countries, including Italy, where there are insufficient doctors and nurses in particular in both intensive and emergency care units in hospitals. Thus, it is necessary to have a health workforce adequately trained and staffed in both the short and long terms, as well as in light of the ageing of the current healthcare professionals, with more effective health workforce planning to guide policy decision-making. This planning could include the enhancement of education opportunities for new doctors and nurses, the improvement of both remuneration and working conditions, and innovation in the overall healthcare supply [19]. Further essential interventions involve the reduction/removal of the architectural barriers of health facilities, which often limit, and even prevent, access to and use of health services, and move towards the concept of universal accessibility, i.e., the elimination of existing obstacles (as already regulated by norms) by adding lacking devices (e.g., lifts) [91]. Another consideration is the utility to provide senior people and their respective caregivers with guidance, e.g., from GPs and social workers, for accessing available health services, and also with adequate support for the whole access process [113]. This would support senior people, especially when facing difficult bureaucratic/administrative practices and procedures for using health services. Also, senior people could be guided in the (self)management of their own chronic conditions, when possible, under the supervision of health professionals and in light of their health status and personal abilities [19].

Public health policies and practices in Italy, as well as worldwide, need to follow the increasing and changing health conditions of senior people, especially in light of lessons learned from the COVID-19 pandemic, to ensure better and sustainable health outcomes [65]. In this respect, both the prevention and early detection of chronic diseases and multimorbidity are crucial steps forward, as indicated in the Italian National Prevention Plan 2020–2025. This could also be achieved by innovating the organisation and infrastructures of local healthcare services [65], with more investments in the resilience of the health system [19] and, subsequently, in “health resilience” for both genders in context of their respective characteristics and peculiarities.

### 4.5. Limitations and Strenghts

This study presents certain limitations that should be considered. The respondents were recruited only from three Italian regions, which are, however, representative of three different levels of socio-economic development in the country, i.e., high in the north (Lombardy region), medium in the centre (Marche region), and low in the south (Calabria region). A simplified definition of older people experiencing frailty was used, namely persons aged 65 years and over with functional limitations and limited mobility, living alone, and needing support for daily activities. Senior people experiencing cognitive challenges were excluded so that only individuals who could answer questions autonomously were selected. To achieve this aim, cognitive status was based on regular assessments carried out by the recruitment channels, and no cognitive test was administered by the interviewers. To calculate the level of functional limitations (self-reported), only daily activities that participants were not able to carry out by themselves were considered. This was to highlight that self-reported functional/physical conditions could include both over- and underestimations of respondents, but the former are considered important in the literature since they can even predict mortality [114]. The purposive sample built for this study allows only typological and not statistically representative findings. Services used in hospitals are also considered (e.g., diagnostic tests), but hospital admission was not included as a separate public health service, since it implies, in turn, the use of diverse/more health services, even though this particular insight could have provided further gendered considerations. Barriers limiting/preventing the use of health services were considered as a whole, and not with precise reference to each one used, even though this further analysis could have provided additional inputs. Moreover, a more in-depth exploration of the mechanism linking barriers and access to health services, by means of further quantitative analyses, could integrate interpretations that were obtained from a mainly qualitative study. Also, a further analysis of results by age groups has not been provided using the small sample, even though this could support possible differences based on age. Finally, several Italian studies were considered, which reduced relevance to a wide international audience. Also, percentage values in the tables need to be interpreted with caution when the respective absolute values are very small. Despite these limitations, a strength of this study is its trustworthiness [115], which is assured by the actions taken to enhance transferability, dependability, confirmability, and credibility. However, member-checking, i.e., the involvement of the participants in this study to check/review and validate the main findings/interpretations drawn from their narratives, was not carried out, so as not to stress/tire the senior interviewees too much [61]. This missing factor, in addition to the missing integration of our results with other sources (e.g., observations, documents) to support our interpretations, did not allow triangulation [61]. Member-checking and triangulation would have supported more confirmability and credibility, respectively.

It is worth considering that our strict criteria and specific purposive sample (older people with intermediate mobility, living alone or with a PCA, and with no help from close relatives) could explain some differences between our findings and those of previous studies on the topic of healthcare use. This sample indeed includes only some typologies of senior people with functional limitations. Also, with the aim of providing a synthetic vision of the topic, the categories which are proposed in Table 1 can appear too simple and generic, without showing the development of additional concepts. However, further strengths of our study include the collection of qualitative interviews/narratives, for which a purposive sample was appropriate, and the reporting of verbatim quotations, which allows us to better examine certain attitudes of the respondents.

## 5. Conclusions

This study, despite its simple/descriptive qualitative approach with a limited sample, aimed to analyse the use of health services by older people experiencing frailty and ageing in place alone in Italy, with a focus on the gender dimension. Findings revealed that women, more than men, reported pathologies, especially arthritis/osteoporosis, and the greater use of drugs. Furthermore, in addition to the use of GPs (reported by all participants of both genders) and of MSs (reported by both genders, mostly private sector), diagnostic tests, especially in the public sector, and nursing care, especially in the private sector, were more commonly used by men, whereas rehabilitation was more commonly reported by women, especially in the private sector. Barriers to using health services are the long waiting lists, particularly in the public sector; the cost of services, only in the private sector; and travel distance in both. Also, apart from the problem of waiting times, which was similarly complained about by both genders, more women than men reported the issue of private medical examinations/treatments being too expensive, especially MS visits, and the need to be accompanied since medical units are often too far away and therefore difficult to reach independently. Multimorbidity and, consequently, polypharmacy and the need for several health services represent crucial challenges for the Italian healthcare system, especially in light of obstacles limiting/preventing this use by older people with functional limitations and frailty. This picture calls into question the necessity of strengthening prevention policies and community services in Italy in order to offer more appropriate, affordable, and timely health services to senior people, taking into consideration the greater intake of arthritis/osteoporosis and the need for rehabilitation for older women, in addition to their greater financial constraints and need for help to cover the travel distance to medical care units. Policies and interventions should be developed for both genders, with a special focus on older females, to eliminate barriers preventing not only their access to health services but also their participation in the labour market and income. This could in turn lead to women’s increased wellbeing, especially later in life [64].

Future research should assess in greater depth the co-existence of multimorbidity and polypharmacy, since these are linked and correlated concepts [116]. Future research could also analyse more in depth the issue of health services used by older people experiencing frailty and ageing in place, focusing both on gender dimension linked to different territorial contexts (e.g., urban/rural and regional), different age groups (e.g., 65–74 years as the youngest-old, 75–84 years as middle-old, and over 85 years as the oldest-old) [117], different households (e.g., senior people living with a spouse/partner, and/or with caring children), and different settings (e.g., at home or in hospital). This could be accomplished by using multiple sources of data, e.g., secondary analysis of health data from a gender perspective, and new gender-disaggregated health data by means of gender-related indicators, in addition to more in-depth qualitative studies [24]. Overall, future research could introduce mixed methods on a larger sample to enhance the interpretability and universality of the results. Such an approach could indeed provide more representative research findings, with a more in-depth assessment of both social and economic determinants of health [65], with the aim of exploring better wellbeing models that are both age- and gender-specific/sensitive, including various social, cultural, and economic factors impacting health [118].

All of the above factors have potential to allow the Italian NHS to meet the growing needs of an increasingly non-self-sufficient older population with chronic diseases [106] and to attain more substantial health equity by addressing the higher levels of multimorbidity among older women. This providing a more personalised and gender-responsive performance [119] with a life-long approach [24]. Importantly, considering gender differences in prevention practices, and in the diagnosis and treatment of illness, in light of precision medicine, should be the primary goal, since such an approach “will benefit men’s and women’s health” [105] (p. 565).

Even though this small study allows for, above all, reflections and confirmations of previous studies for a general discussion on the topic, the results highlight certain female-specific factors which also emerged from previous studies, i.e., higher morbidity rates, higher healthcare service utilisation frequencies, and more significant structural barriers in accessing and using services themselves, which can represent a reinforced reference point for formulating more targeted care policies for older people, also in terms of the gender dimension. This study thus has a positive significance for advancing the construction of an age-friendly health service system and could inform healthcare practices and policy, thus being useful for health professionals and policymakers, with the aim of stressing the need to promote equity in care.

## Figures and Tables

**Table 1:** Categories and coding.

Macro-Categories	Sub-Categories	Coding
Health	Diseases	Arthritis/osteoporosis; heart disease/hypertension; diabetes; asthma/chronic obstructive pulmonary disease (COPD); cancer; Parkinson’s; anxiety/depression; urinary incontinence Gender: female, male
	Use of drugs	Number per day: 2–4 ^1^, 5–7, 8+, several/not specified Gender: female, male
Health services used	Public health services	General practitioner (GP); medical specialist (MS); diagnostic tests ^2^; rehabilitation; nurse ^3^ Gender: female, male
	Private health services	Medical specialist (MS); diagnostic tests ^2^; rehabilitation; nurse ^3^ Gender: female, male
Barriers for using services	Public health services	Long waiting lists; transport/distance; architectural barriers; bureaucracy; poor treatment; no home visits by GP Gender: female, male
	Private health services	High cost; transport/distance; long waiting lists; architectural barriers; bureaucracy Gender: female, male

^1^ Coding of drugs, as number of used drugs for indicating the intensity per day of the assumption, starts from “2” because nobody reported the use of only one drug; ^2^ Diagnostic tests in medical units, e.g., blood/urine tests, ultrasound, electrocardiogram (ECG); ^3^ Nurses at home for injections, blood drawing, and catheter management.

**Table 2:** Sample characteristics by gender (n = absolute values).

Characteristics	Female		Male		Total	
	n	%	n	%	n	%
**Age Groups** (**years**)						
67–79	25	28	11	36	36	30
80 and over	65	73	19	63	84	70
**Education**						
No title (no educational qualification/no formal schooling) ^1^	11	12	3	10	14	12
Primary/Middle School (5 and 3 years)	55	61	20	66	75	63
High School/University (3–5 years both)	24	26	7	23	31	25
**Marital Status**						
Single	11	12	5	17	16	13
Divorced/separated ^2^	6	7	10	33	16	13
Widowed	73	81	15	50	88	73
**Living Situation**						
Alone	72	80	21	70	93	78
With personal/private care assistant (PCA)	18	20	9	30	27	22
**Level of physical/functional limitations ^3^**						
Mild/Moderate	53	59	10	33	63	53
High/Very High	37	41	20	67	57	47
**Mobility**						
Only/mainly in the home ^4^	34	38	14	47	48	40
Also (more frequently) outside the home with help ^5^	56	62	16	53	72	69
**Total respondents**	90	100	30	100	120	100

^1^ This category includes persons who have not obtained any certificates, diplomas, or degrees or equivalent. ^2^ This includes two male respondents still married but not cohabiting with their spouses (de facto separated). ^3^ The level of physical/functional limitations is based on 12 Basic and Instrumental Activities of Daily Living (ADLs–IADLs), two mobility limitations (going up/down the stairs and bending to pick up an object), plus sensory limitations in hearing and eyesight. Mild = no activities “not able”; Moderate = one–two; High = three–four; Very high = five or more. ^4^ This includes also respondents able to move outside the home very rarely, i.e., less than two times a week and only if accompanied or with aids (cane, walker). ^5^ Respondents are able to move within the home and also outside at least two times a week, only if accompanied or with aids (cane, walker).

**Table 3:** Main diseases and use of drugs by gender (n = number of statements).

Diseases and Drugs ^1^	Female		Male		Total	
Diseases ^2^	n	%	n	%	n	%
Arthritis/Osteoporosis	66	73	11	37	77	64
Heart Disease/Hypertension	30	33	10	33	40	33
Diabetes	12	13	4	13	16	13
Cancer ^3^	12	13	2	7	14	12
Anxiety/Depression	11	12	3	10	14	12
Asthma/Chronic Obstructive Pulmonary Disease (COPD) ^4^	10	11	3	10	13	11
Urinary Incontinence	8	9	2	7	10	8
Parkinson	2	2	-	-	2	2
**Number of drugs per day ^5^**	n	%	n	%	n	%
2–4 ^6^	20	22	9	30	29	24
5–7	18	20	8	27	26	22
8+	14	16	4	13	18	15
Several/Not Specified	30	33	7	23	37	31
**Total respondents**	90	100	30	100	120	100

^1^ Percentages are calculated for total respondents (90 females, 30 males, 120 total). ^2^ Some respondents reported more than one type of listed diseases. Problems with hearing and seeing were included in the analysis of the functional limitations, and thus are not reported here. ^3^ Including cancer episodes resolved at the time of interview. ^4^ Including general seroius breathing problems. ^5^ Some respondents did not report any drug use. ^6^ Nobody reported the use of only one drug.

**Table 4:** Older people using public and private health services by gender (n = number of statements).

Health Services ^1^	Female		Male		Total	
Public Health Services ^2^	n	%	n	%	n	%
General Practitioner (GP)	90	100	30	100	120	100
Other health services ^3^	53	59	18	60	71	59
**Private health services ^3^**	52	58	14	47	66	55
**Both public and private** (excluding GP, used by all)	29	32	6	20	35	29
**Total respondents**	90	100	30	100	120	100

^1^ Percentages are calculated for total respondents (90 females, 30 males, 120 total). Some respondents did not report any use (apart from GP, used by all), and, conversely, some respondents reported the use of services in both sectors; ^2^ Two main “Public health services” macro-groups are indicated to highlight the use of GPs by all respondents, when compared to other public health services. In “Private health services” all relevant services are included/grouped. In Table 5 below, both public (apart from GPs) and private health services are listed/specified; ^3^ Medical specialists (MSs), diagnosric tests, rehabilitation, nurses.

**Table 5:** Main public and private health services used by type and gender (n = number of statements).

Health Services ^1^	Female		Male		Total	
Public Health Services (Apart From GP)	n	%	n	%	n	%
Medical specialist (MS) ^2^	36	68	12	67	48	68
Diagnostic tests ^3^	11	21	5	28	16	23
Rehabilitation	8	15	3	17	11	15
Nurse ^4^	7	13	1	6	8	11
**Respondents who use public health services**	53	100	18	100	71	100
**Private Health Services**	**n**	**%**	**n**	**%**	**n**	
Medical specialist (MS) ^2^	41	79	11	79	52	79
Nurse ^4^	14	27	5	36	19	29
Rehabilitation	13	25	2	14	15	23
Diagnostic tests ^3^	3	6	1	7	4	6
**Respondents who use private health services**	52	100	14	100	66	100

^1^ Percentages are calculated only on respondents who reported to use, respectively, public, and private health services. Some respondents reported more than one type of listed public and private health services; ^2^ MSs were mostly cardiologists, physiatrists, orthopedists, and diabetologists; ^3^ diagnostic tests (in medical units) were mostly blood/urine tests, ultrasounds, electrocardiograms (ECG), and computed axial tomography (TAC); ^4^ Nurses visited at home for injections, blood drawing, and catheter management.

**Table 6:** Main barriers for using public and private health services by gender (n = number of statements).

Barriers ^1^	Female		Male		Total	
Public Health Services (Including GP)	n	%	n	%	n	%
Long waiting lists	18	20	8	27	26	22
Transport/distance ^2^	20	22	1	3	21	18
Architectural barriers ^2^	5	6	2	7	7	6
No home visit by GP	5	6	2	7	7	6
Bureaucracy	4	4	-	-	4	3
Poor treatment ^2^	2	2	2	7	4	3
**Private Health Services**	**n**	**%**	**n**	**%**	**n**	**%**
High cost	23	26	6	20	29	24
Transport/distance	12	13	2	7	14	12
Long waiting lists	8	9	1	3	9	8
Architectural barriers	2	2	-	-	2	2
Bureaucracy	1	1	-	-	1	1
**Total respondents**	90	100	30	100	120	100

^1^ Percentages are calculated on total respondents (90 females, 30 males, 120 total), since barriers were also reported by those who do not use health services (apart from GPs, used by all). However, some respondents reported more than one type of listed barriers, and some respondents did not report any. ^2^ These barriers were reported also with regard to GPs.

## Data Availability

All relevant data analysed during this study, and supporting the findings (i.e., absolute values, relevant quotations), are within the manuscript. A minimum dataset with quantitative data presented in this study (e.g., socio-demographic) are openly available in Mendeley at https://doi.org/10.17632/m4jrt9snhv.1 (accessed on 13 June 2025). The qualitative dataset, i.e., charts containing the verbatim transcripts of narratives (in Italian), are not publicly available due to privacy/ethical restrictions, since there are personal/sensitive information, e.g., names and locations of persons, that could compromise the anonymity of older respondents.

## Supplementary Materials

The following supporting information can be downloaded at: https://www.mdpi.com/article/10.3390/healthcare13212684/s1. File S1: Checklist. COREQ—Consolidated criteria for REporting Qualitative research.
